# The Floating Threat: A Rare Case Report of Carotid Saddle Thrombus in a Healthy Adult

**DOI:** 10.5811/cpcem.48938

**Published:** 2026-05-23

**Authors:** Ariel Droger, Rolando Torres-Castro, Kashan Mahmood, Jason Graf, Sean Serio, Alexander John Scumpia

**Affiliations:** HCA FL Aventura Hospital, Department of Emergency Medicine, Aventura, Florida

**Keywords:** free-floating thrombus, saddle embolus, stroke, carotid artery, anticoagulation

## Abstract

**Introduction:**

Carotid free-floating thrombus is a rare and potentially devastating cause of ischemic stroke. Diagnosis remains challenging due to the dynamic nature of the lesion.

**Case Report:**

We report the case of a 46-year-old female presenting with neck pressure and gait instability, who was found to have a free-floating thrombus at the brachiocephalic-carotid junction. Despite early anticoagulation, she developed biparietal ischemic strokes.

**Conclusion:**

This case highlights the challenges in management of carotid free-floating thrombus including appropriate anticoagulation, contraindications to thrombolysis, and the need for multidisciplinary involvement. Emergency physicians must maintain high suspicion for vascular pathology in atypical neurologic presentations and recognize that even optimal medical therapy does not eliminate stroke risk.

## INTRODUCTION

Carotid free-floating thrombus is a rare clinical finding characterized by a thrombus attached to the arterial wall without causing complete occlusion.^1^ While typically more prevalent in males and younger populations compared to other carotid pathologies,^4^ their incidence is likely under-recognized, with improved detection attributed to the growing use of non-invasive vascular imaging.^15^ Incidence of carotid free-floating thrombus with carotid disease, strokes, or transient ischemic attack (TIA) is approximately 0.6–2.5%. This figure reflects the rarity and varying incidence of this condition based on the population studied and diagnostic methods used. True incidence in the general population is likely lower as most data are derived from selected cohorts of stroke and carotid intervention patients. Carotid free-floating thrombus remains an uncommon but clinically significant cause of stroke.^3^,^17^ The diagnosis remains challenging due to the dynamic nature of the lesion. Despite increasing numbers of carotid free-floating thrombus being identified, standardized treatment guidelines are lacking; current management strategies are based predominantly on case reports and small case series.^7^,^10^,^12^,^14^,^19^ Treatments range from anticoagulation therapy alone to surgical options such as thromboendarterectomy or carotid artery stenting.^2^ The majority of symptomatic cases are attributed to atherosclerotic plaque rupture; however, other etiologies include hypercoagulable states, endothelial injury, trauma, substance use, dissection, cardioembolism, malignancy, or idiopathic causes.^2^,^4^,^11^

While digital subtraction angiography has been proposed as the gold standard for detection, non-invasive imaging modalities like computed tomography angiography (CTA) and magnetic resonance angiography (MRA) are increasingly being used.^3^ Patients commonly present with TIAs or stroke-like symptoms, often due to microembolization rather than complete thrombus migration.^4^ The internal carotid artery is the most frequently involved vessel, although carotid free-floating thrombus has been reported in other supra-aortic vessels including the common carotid, brachiocephalic, and subclavian arteries.^4^,^5^,^8^

## CASE REPORT

A 46-year-old female with a history of daily tobacco use presented with right-sided head and neck pressure, gait instability, and a history of recent dental work complicated by toothache and steroid injections. Physical examination revealed a rightward gait deviation and positive Romberg sign, with otherwise normal speech, strength, and sensation. Vital signs were stable.

Computed tomography angiography imaging revealed a 1-cm free-floating saddle thrombus at the junction of the left common carotid artery and brachiocephalic trunk (Image). Laboratory testing showed an isolated leukocytosis with a white blood cell count of 18.5 ×10^9/liter (L) (reference range: 4.5–11.0 x 10^9/L). Consultations with vascular surgery, neurointerventional radiology, and neurology resulted in a consensus for anticoagulation with heparin bolus and infusion, intensive care unit admission, permissive hypertension, and hourly neurologic checks.

Several hours after initiation of heparin, the patient developed acute left hemiplegia and hemineglect, rightward gaze deviation, dysarthria, and sensory deficits with a cumulative National Institutes of Health Stroke Scale of 13. This stroke occurred with heparin at supratherapeutic levels, 109–150 seconds, as partial thromboplastin time goal was 80–100 seconds per neurointerventional radiology. Repeat CTA showed hypoperfusion of the watershed zones between the right anterior and posterior cerebral circulation without evidence of residual thrombus. Magnetic resonance imaging confirmed biparietal ischemic strokes without hemorrhagic transformation. Thrombolysis with tissue plasminogen activator (tPA) was contraindicated due to therapeutic anticoagulation with heparin. Transthoracic and transesophageal echocardiography excluded cardioembolic sources; and no patent foramen ovale, left atrial appendage thrombus, infective endocarditis, or valvular vegetations were identified. Cerebral angiography revealed acute ischemic changes without mycotic aneurysms.

*CPC-EM Capsule*

What do we already know about this clinical entity?

*Carotid free-floating thrombus is a rare but high-risk cause of ischemic stroke with no standardized treatment guidelines.*

What makes this presentation of disease reportable?

*This patient developed bilateral ischemic strokes despite supratherapeutic heparin for a rare carotid saddle thrombus.*

What is the major learning point?

*Stroke risk remains high in carotid free-floating thrombus despite early anticoagulation and multidisciplinary care.*

How might this improve emergency medicine practice?

*Emergency physicians should suspect vascular pathology in atypical neurologic symptoms and obtain early vascular imaging.*

Further workup revealed positive lupus anticoagulant antibody (Ab) but negative antinuclear antibodies, double stranded DNA Ab, beta-2 glycoprotein immunoglobulin G Ab, anticardiolipin Ab, and normal homocysteine levels. Of note, the patient was on heparin at the time, which could potentially cause a false positive lupus anticoagulant Ab. Lumbar puncture showed elevated myelin basic protein at 11.3 nanograms per milliliter (ng/mL) and was largely unremarkable with no oligoclonal bands, negative for Venereal Disease Research Laboratory, *Borrelia burgdorferi* and *Cryptococcus*. The patient improved gradually with heparin anticoagulation and intensive rehabilitation. Seven days after the acute ischemic event, the patient was walking with the help of family at bedside with significantly improved left lower extremity weakness and with near full recovery of ability to perform activities of daily living. She was discharged on apixaban for six months with plans for outpatient hypercoagulability evaluation.

## DISCUSSION

This case illustrates a rare instance of carotid free-floating thrombus leading to bilateral ischemic strokes despite appropriate anticoagulation. Most cases are associated with carotid atherosclerotic disease; however, absence of flow-limiting stenosis in this patient necessitated evaluation for alternative causes.^2^,^4^,^11^ Management of carotid free-floating thrombus remains controversial. Successful treatment has been achieved with both anticoagulation and surgical interventions such as thromboendarterectomy or stenting.^4^,^5^,^9^,^17^ No clear consensus exists regarding superiority, although some studies report lower recurrence rates with surgery in selected cases.^17^

In emergency settings, rapid imaging (CTA or MRA) is crucial for patients presenting with atypical neurologic symptoms and neck pain. Duplex ultrasound may miss proximal or saddle lesions.^3^ The decision to withhold tPA was appropriate in this case as American Heart Association guidelines contraindicate thrombolysis in patients on therapeutic heparin with an elevated activated partial thromboplastin time, due to increased hemorrhagic risk. Emergency physicians must involve vascular, neurology, and neurointerventional teams early, even in cases without carotid stenosis, given the embolic risk of carotid free-floating thrombus.

This case reinforces that despite appropriate medical therapy, stroke risk remains high in carotid free-floating thrombus patients, particularly in the first week after diagnosis.^11^,^19^ Intensive care unit monitoring with permissive hypertension and neurologic surveillance is recommended. Ultimately, management should be multidisciplinary and individualized based on thrombus characteristics, imaging findings, and patient comorbidities.

## CONCLUSION

We present a rare case of a free-floating carotid thrombus in a 46-year-old female who developed biparietal strokes despite proper anticoagulation, with gradual return to near-full recovery. This case illustrates the importance of early imaging, multidisciplinary management, and recognition of contraindications to thrombolysis in the emergency department.

## Figures and Tables

**Image.**
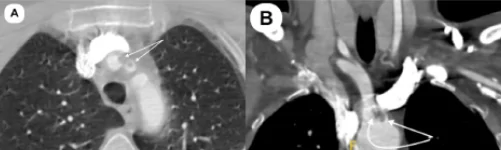
Computed tomography angiogram of the head and neck, (A) axial view and (B) coronal view, demonstrating a free-floating thrombus (arrows) at the junction of the left common carotid artery and brachiocephalic trunk.

## References

[1] Cancer-Perez S, Alfayate-García J, Vicente-Jiménez S (2021). Symptomatic common carotid free-floating thrombus in a COVID-19 patient: case report and literature review. Ann Vasc Surg.

[2] Shiozaki E, Morofuji Y, Kawahara I (2021). Free-floating thrombus in the carotid artery without atherosclerosis dissolved by antithrombotic therapy. Neurol India.

[3] Ferrero E, Ferri M, Viazzo A (2011). Free-floating thrombus in the internal carotid artery: diagnosis and treatment of 16 cases in a single center. Ann Vasc Surg.

[4] Bhatti AF, Leon LR, Labropoulos N (2007). Free-floating thrombus of the carotid artery: literature review and case reports. J Vasc Surg.

[5] Oki N, Inoue Y, Kotani S (2021). Free-floating thrombus of the aorta: 3 case reports. Surg Case Rep.

[6] Yang P, Li Y, Huang Y (2020). A giant floating thrombus in the ascending aorta: a case report. BMC Surg.

[7] Christou N, Gourgiotis I, Dakis K (2021). Embolic strokes in a patient with a large floating thrombus in the ascending aorta. Hippokratia.

[8] Torrealba JI, Valdés FJ, Garrido L (2022). Acute symptomatic free-floating thrombus in the innominate artery: a case series. Vasc Endovascular Surg.

[9] Chang H, Rockman CB, Narula N (2022). Presentation, diagnosis and management of innominate artery thromboembolism. J Endovasc Ther.

[10] Jayyusi F, AlBarakat MM, Al-Rousan HH (2024). The efficacy of medical interventions for free-floating thrombus in cerebrovascular events: a systematic review. Brain Sci.

[11] Dowlatshahi D, Lum C, Menon BK (2023). Aetiology of extracranial carotid free-floating thrombus in a prospective multicentre cohort. Stroke Vasc Neurol.

[12] Bouchal S, Essayeh G, Naouli H (2023). Recurrent floating common carotid artery thrombus related to COVID-19: a case report. J Med Vasc.

[13] Vassileva E, Daskalov M, Stamenova P (2015). Free-floating thrombus in stroke patients with nonstenotic internal carotid artery: an ultrasonographic study. J Clin Ultrasound.

[14] Gülcü A, Gezer NS, Men S (2014). Management of free-floating thrombus within the arcus aorta and supra-aortic arteries. Clin Neurol Neurosurg.

[15] Lane TR, Shalhoub J, Perera R (2010). Diagnosis and surgical management of free-floating thrombus within the carotid artery. Vasc Endovascular Surg.

[16] Müller MD, Raptis N, Mordasini P (2022). Natural history of carotid artery free-floating thrombus: a single center, consecutive cohort analysis. Front Neurol.

[17] El Harake S, Doche E, Bertolino J (2023). Symptomatic carotid free-floating thrombus: management of 50 cases in a referral neurovascular center. J Clin Med.

[18] Kelley JD, Kerndt CC, Ashurst JV (2024). Anatomy, thorax, aortic arch. StatPearls [Internet].

[19] Fridman S, Lownie SP, Mandzia J (2019). Diagnosis and management of carotid free-floating thrombus: a systematic literature review. Int J Stroke.

